# Small GTPase Rap1 Is Essential for Mouse Development and Formation of Functional Vasculature

**DOI:** 10.1371/journal.pone.0145689

**Published:** 2015-12-29

**Authors:** Magdalena Chrzanowska-Wodnicka, Gilbert C. White, Lawrence A. Quilliam, Kevin J. Whitehead

**Affiliations:** 1 Blood Research Institute, BloodCenter of Wisconsin, Milwaukee, WI, 53201, United States of America; 2 Department of Biochemistry and Molecular Biology, Indiana University School of Medicine, Indianapolis, IN, 46202, United States of America; 3 Division of Cardiovascular Medicine, Pediatric Cardiology, Molecular Medicine Program, University of Utah, Salt Lake City, UT, 84112, United States of America; Yale University School of Medicine, UNITED STATES

## Abstract

**Background:**

Small GTPase Rap1 has been implicated in a number of basic cellular functions, including cell-cell and cell-matrix adhesion, proliferation and regulation of polarity. Evolutionarily conserved, Rap1 has been studied in model organisms: yeast, Drosophila and mice. Mouse *in vivo* studies implicate Rap1 in the control of multiple stem cell, leukocyte and vascular cell functions. *In vitro*, several Rap1 effectors and regulatory mechanisms have been proposed. In particular, Rap1 has been implicated in maintaining epithelial and endothelial cell junction integrity and linked with cerebral cavernous malformations.

**Rationale:**

How Rap1 signaling network controls mammalian development is not clear. As a first step in addressing this question, we present phenotypes of murine total and vascular-specific *Rap1a*, *Rap1b* and double *Rap1a* and *Rap1b* (Rap1) knockout (KO) mice.

**Results and Conclusions:**

The majority of total Rap1 KO mice die before E10.5, consistent with the critical role of Rap1 in epithelial morphogenesis. At that time point, about 50% of Tie2-double Rap1 KOs appear grossly normal and develop normal vasculature, while the remaining 50% suffer tissue degeneration and show vascular abnormalities, including hemorrhages and engorgement of perineural vessels, albeit with normal branchial arches. However, no Tie2-double Rap1 KO embryos are present at E15.5, with hemorrhages a likely cause of death. Therefore, at least one Rap1 allele is required for development prior to the formation of the vascular system; and in endothelium–for the life-supporting function of the vasculature.

## Introduction

The evolutionarily conserved and ubiquitously expressed small GTPase Rap1 is at the crossroads of signaling pathways that govern key cellular processes. Downstream from multiple receptors, Rap1 activity is spatiotemporally regulated by a network of guanine nucleotide exchange factors (GEFs) and GTPase activating proteins (GAPs) acting in a tissue-specific manner [1]. In a GTP-bound form, activated Rap1 interacts with a number of effectors to control cell-substrate adhesion, cell-cell adhesion and junction formation [2], and cell polarity [3]. *In vitro* studies have implicated KRIT1/CCM1, a protein mutated in cerebral cavernous malformations (CCM), RASIP1 and an actin-cytoskeleton linker Canoe/Afadin as Rap1 effectors controlling cell-cell junction formation and maintenance.

In particular, Rap1 interaction with KRIT1 [4–7] has raised interest due to its potential significance for human disease [8]. KRIT1, one of three proteins whose autosomal mutations have been linked with CCM; a neurovascular malformation syndrome that leads to seizures and lethal stroke [9–12], is a multi-domain protein that links cortical actin cytoskeleton with integral membrane proteins, and interacts with CCM2 [13]. *In vitro*, in endothelial cells (ECs), Rap1 facilitates localization of KRIT1 to cell-cell junctions and interaction with junctional proteins [5, 14, 15]. However, whether Rap1-KRIT1 interaction plays a physiological role in the development of CCM is unknown.

Rap1 functions *in vivo* have been studied in several model organisms. In lower organisms, a single Rap1 ortholog plays a central role in the development of cell polarity: in budding yeast *S*. *cerevisiae*, Rap1 ortholog Bud1/Rsr controls positioning of the bud [16] and in Drosophila, Rap1 controls apico-basal polarity during mesoderm formation and dorsal closure via its interactions with a protein network involving atypical PKC (aPKC) and Canoe/Afadin [17–19]. In higher organisms, two highly homologous isoforms of Rap1 exist: Rap1a and Rap1b. The two Rap1 isoforms are encoded by separate genes [20, 21] and murine genetic deletion models of both have been described [8]. Deletion of either isoform leads to partial embryonic lethality and bleeding [22, 23]. While deletion of either Rap1 isoform does not limit the lifespan of surviving adult mice, several defects in neurological [24] and immune responses [23, 25–28] and hematopoiesis [29] have been described. Some of the most significant defects observed in Rap1 knockout (KO) mice involve their cardiovascular functions: platelet function [22], angiogenesis [30, 31], smooth muscle contractility and vessel tone [32]. The similarity of some of the phenotypes of the two Rap1 isoforms KOs suggested functional redundancy. To determine if the two isoforms have similar functions, we attempted to generate double *Rap1a*, *Rap1b* KO mice. In this paper, for the first time to our knowledge, we report on the phenotype of these mice.

Because of the bleeding phenotype in total Rap1-deficient embryos we have been particularly interested in the role of Rap1 in the vasculature. We have made endothelial lineage restricted Rap1 KO mice and demonstrated a critical role of Rap1 in endothelial cells in angiogenesis [32, 33] and, more recently, regulation of endothelial function and blood pressure [34]. Interestingly, molecular mechanisms underlying these defects in adult mice implicate Rap1 in regulation of the signaling aspect of adhesion receptors [32, 33], rather than in their role in promoting cell adhesion. However, the role of Rap1 in vasculature during morphogenesis and development is not known. To address this knowledge gap, here we describe the phenotype of Rap1 endothelial-specific KO mice and analyze it in the context of Rap1 effectors, KRIT1 and Afadin.

## Methods

### Animal generation and husbandry

All mouse procedures were performed according to approved Medical College of Wisconsin or Indiana University School of Medicine Institutional Animal Use and Care Committee protocols. Generation of *Rap1b* ^*-/-*^ mice and endothelial-specific Rap1 KO mice (EC-Rap1 KO; Tie2-Cre^+/0^ Rap1a^f/f^ Rap1b^f/f^) (with a mixed 129SvEv/C57BL6, 50%/50% average, background) has been previously described [22, 33]. *Rap1b* ^*+/-*^ mutant mice were back-crossed with C57BL/6 mice (The Jackson Laboratory, Bar Harbor, ME, USA) for at least 8 successive generations. Double KO *Rap1a* ^*-/-*^ *Rap1b* ^-/-^ mice were generated by intercrossing *Rap1b* ^*-/-*^ with *Rap1a* ^*-/-*^ mice [23]. Mouse genotypes were determined by PCR on tail DNA as described previously [22, 23, 33].

### Determination of embryonic lethality

Timed pregnancies were set up with mid-day of vaginal plug defined as E0.5. Embryos were collected at specified times and genotypes were determined by PCR of tissue samples. Expected embryo numbers (N_E_) were determined using Mendelian ratios, based on 100% survival of WT embryos. Embryo survival was defined as observed (N_O_) vs. expected (N_O_/N_E_*100%); embryo lethality was defined as (1-N_O_/N_E_)*100%.

### Histology

Embryos were collected and bright-field images were obtained using Zeiss stereoscope (SteREO Lumar.V12, Carl Zeiss MicroImaging GmbH, Germany) at 6.4x magnification. Histological staining was performed as previously described [35, 36]. Briefly, paraffin sections were stained with hematoxilin and eosin (H&E) or with antibodies to CD31 (PECAM-1, at 1:250 dilution, clone MEC13.3, BD Biosciences). Improved visualization on paraffin sections was obtained using a biotinylated tyramide signal amplification (TSA) kit (PerkinElmer) according to the manufacturer’s instructions.

## Results

### Partial embryonic lethality of total *Rap1b* KO mice

Two highly homologous Rap1 isoforms exist encoded by separate *Rap1a* and *Rap1b* genes [20, 21]. We reported partial embryonic and perinatal lethality of *Rap1b* ^-/-^ mice on mixed background [22]. While *Rap1b* ^-/-^ females (on mixed genetic background) were fertile, litter size was reduced (4 ± 0.28, s.e.m., surviving pups/litter; n = 40 litters from *Rap1b* ^*-/-*^ intercrosses) compared to WT litter size (7.2 ± 0.37, s.e.m., surviving pups/litter; n = 40 litters from *Rap1b* ^*+/+*^ intercrosses). In about 20% of embryos, starting at E13.5 we observed dispersed subcutaneous bleeding, and hemorrhages on the side of the head and in liver, accompanied by edema [22]. To better determine the time and cause of embryo lethality, we performed systematic analysis of *Rap1b* ^-/-^ embryos from staged pregnancies. We found that at E13.5 and E15.5, *Rap1b* ^-/-^ embryo number (on mixed genetic background) was not reduced compared to WT embryos (80–90 embryos analyzed), however about 30–50% *Rap1b* ^-/-^ embryos appeared abnormal, containing hemorrhages or clots. At E18.5 about 20% of *Rap1b* ^-/-^ embryos were found resorbing. Furthermore, we found *Rap1b* ^-/-^ embryo body weight significantly decreased at E15.5 (0.233 ± 0.013 vs. 0.273 ± 0.010 g, WT; p<0.05) and at E18.5, (1.119 ± 0.036 g vs. 1.242 ± 0.030 g, WT; p<0.05). The difference in body weight persisted after birth [32]. Observed growth restriction of embryos is consistent with a systemic defect, such as in vascular development, and may contribute to decreased viability at weaning to 28% of expected numbers [22], (Table 1).

**Table 1:** Survival of *Rap1b* ^-/-^ and *Rap1a* ^*-/-*^ mice at weaning.

	*Mixed C57Bl6/129SvEv background*				*C57Bl6 background*				*C57Bl6 background*			
Genotype	WT	Rap1b^+/-^	Rap1b^-/-^	Total	WT	Rap1b^+/-^	Rap1b^-/-^	Total	WT	Rap1a^+/-^	Rap1a^-/-^	Total
Mice at weaning	357	667	100	1124	392	522	12	926	92	188	59	339
*Expected* */	*357*	*714*	*357*		*392*	*784*	*392*		*92*	*184*	*92*	
Viable/Expected		93%	28%			67%	3%			≥100%	64%	

Number of adult mice from *Rap1b* ^*+/-*^ or *Rap1a* ^*+/-*^ intercrosses on mixed and C57Bl6 backgrounds.

*/Expected number = Total number * Mendelian frequency (1:2:1), based on 100% survival of WT mice.

**Fig 1:**
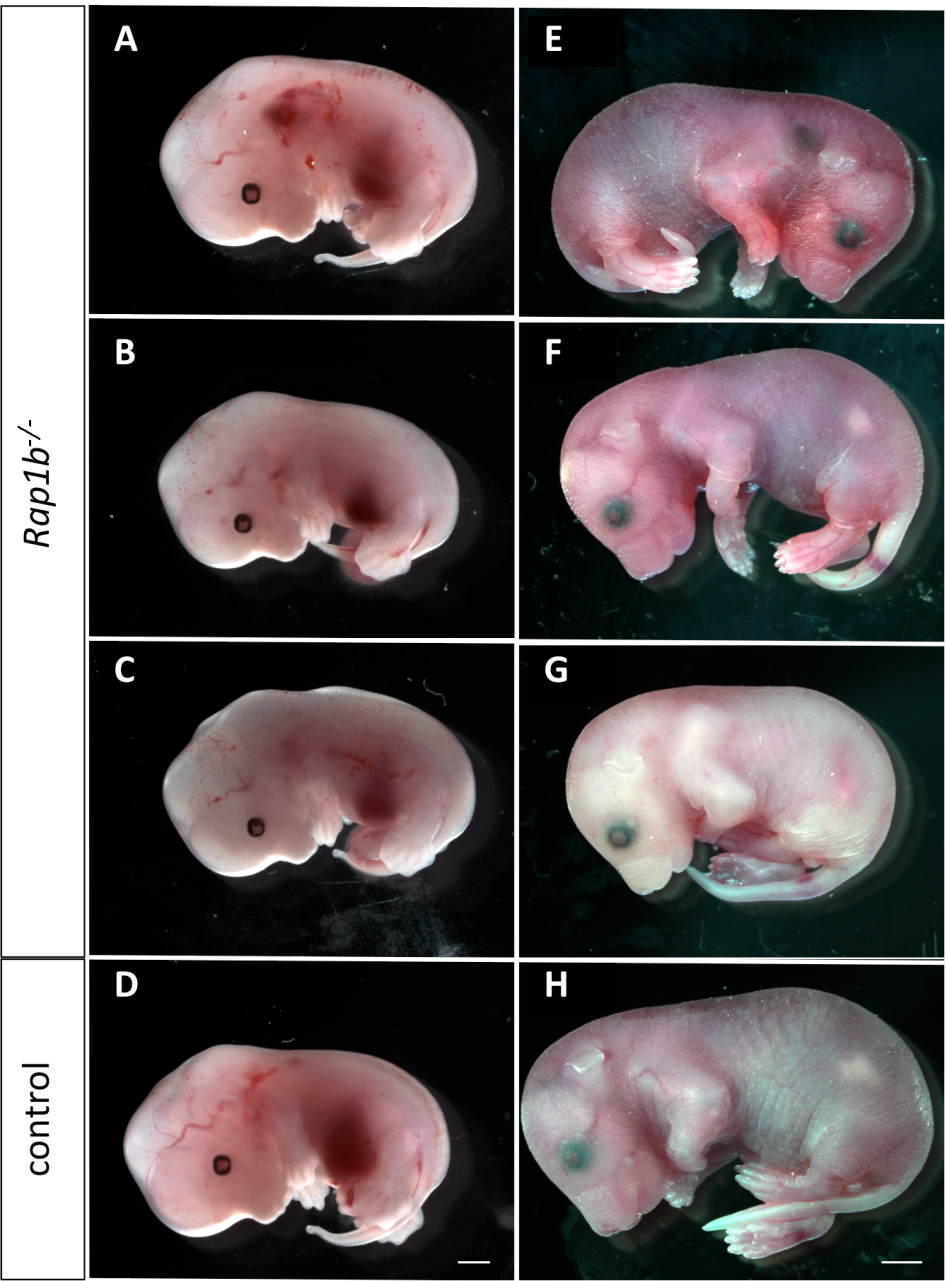
Vascular abnormalities in *Rap1b* ^*-/-*^ embryos on C57Bl6 genetic background. Embryos from pregnancies resulting from *Rap1b* ^*+/-*^ intercrosses at E13.5 (A-D, left panel) and E18.5 (E-H, right panel) on C57/BL6 genetic background. Rap1b-deficiency leads to cranial hemorrhage in about 20–50% of embryos (A, E), edema (C), pale pallor (G), and a smaller body size compared to WT littermate controls (D, H). Stereoscopic images are representative of 4–6 analyzed pregnancies. Scale bars: 1 mm (A-D) and 2 mm (E-H).

Initial observations of *Rap1a* ^*-/-*^ mice on mixed genetic background did not suggest lethality, however upon backcross onto C57Bl6 background (F7-11), partial embryonic lethality occurred [23], see Table 1. We hypothesized that *Rap1b* ^-/-^ mice on pure C57Bl6 background may reveal additional phenotype that may not be apparent in the mixed background due to modifier genes. Upon back-crossing *Rap1b* ^-/-^ mice to F8 onto C57Bl6 background we examined survival of *Rap1b* ^-/-^ mice. We found further decreased survival of mice at weaning (Table 1). To determine the time of embryonic death, we examined homozygous KO embryos from staged pregnancies (Fig 1). No overt morphological defects were seen before E10.5 *Rap1b* ^-/-^; however, at that time, *Rap1b* ^*-/-*^ embryos were smaller in size (Fig 1A).

At E13.5 and E15.5, about 20–50% of *Rap1b* ^*-/-*^ embryos on C57Bl6 background displayed prominent hemorrhage on the side of the head, indicative of cardiovascular defects, while other *Rap1b* ^*-/-*^ had no overt defects (Fig 1B). Similarly, at E18.5 (Fig 1C) this bleeding phenotype was only displayed in about 25% of *Rap1b* ^*-/-*^ embryos. With 97% lethality at weaning (Table 1), we conclude that most *Rap1b* ^*-/-*^ mice die perinatally and Rap1b is not absolutely required for embryonic development.

### Early embryonic lethality of *Rap1a*, *Rap1b* KO mice

**Fig 2:**
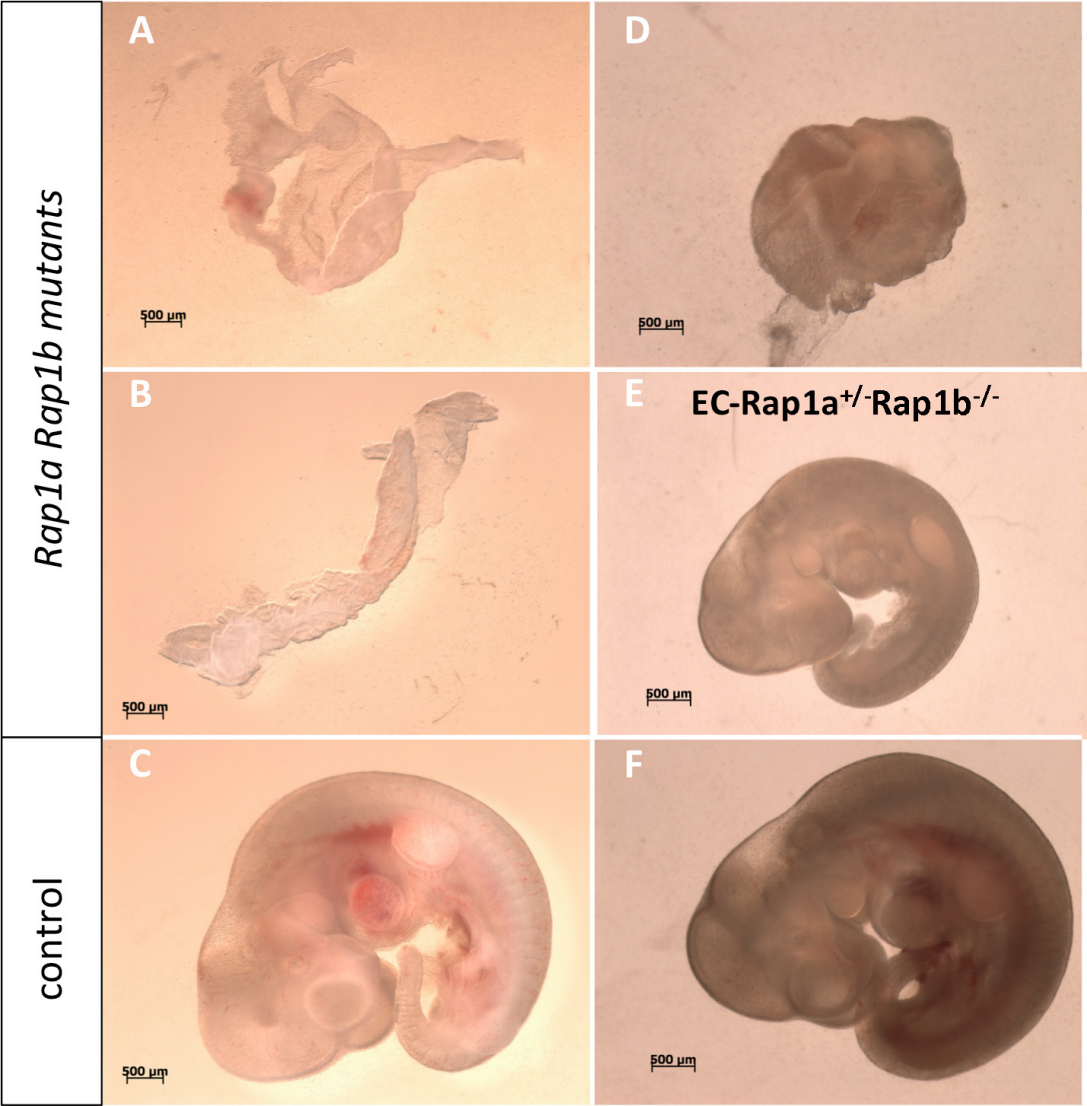
Double KO of Rap1a and Rap1b leads to lethality of majority of embryos before E10.5. Stereoscopic images from two (A-C; D-F) of eight analyzed pregnancies resulting from *Rap1a* ^*+/-*^, *Rap1b* ^*+/-*^ intercrosses. About 80% of *Rap1a* ^*-/-*^ *; Rap1b* ^*-/-*^ embryos (A, B, D) fail to develop and pregnancy products are grossly malformed and non-viable. (E) Deletion of both *Rap1b* and one *Rap1a* allele leads to severe growth restriction; (C, F) littermate *Rap1a* ^*+/-*^, *Rap1b* ^*+/+*^ controls. Scale bar: 0.5 mm.

In lower organisms Rap1 is critical for morphogenesis [18] and deletion of the only isoform of Rap1 present in these organisms leads to lethality [37]. Since deletion of either *Rap1a* or *Rap1b* genes is not essential for development, as some KOs survive to adulthood, we hypothesized that there is a redundancy in Rap1a and Rap1b function in vertebrates. To test if either Rap1 gene is essential for development, we attempted to generate a double *Rap1a*, *Rap1b* KO (“Rap1 KO”) by intercrossing *Rap1a* ^*+/-*^ and *Rap1b* ^*+/-*^ mice, but no viable Rap1 KO mice were obtained (Table 2). We therefore examined the effect of Rap1 deletion on embryonic development. We found that at E8.5 all expected embryos were recovered and appeared grossly normal, which indicates that Rap1 is not absolutely required for early development. However at E10.5, a reduced number of Rap1 KO embryos was recovered and only about 20% Rap1 KO embryos appeared viable (Table 3), while the remaining 80% of Rap1 KO embryos appear grossly underdeveloped and abnormal (Fig 2). Thus, we conclude that, unlike in lower organisms [38], Rap1 in mice is not critical for early embryonic events, such as gastrulation; but it is required for further development.

**Table 2:** Survival of offspring from *Rap1a* ^*+/-*^,*Rap1b* ^*+/-*^ intercrosses,(on mixed genetic background).

Genotype	Total	Expected */	Viable/Expected
Rap1a^+/+^Rap1b^+/+^	13	13	100%
Rap1a^+/+^ Rap1b^+/-^	32	26	≥100%
Rap1a^+/+^ Rap1b^-/-^	6	13	46%
Rap1a^+/-^ Rap1b^+/+^	47	26	≥100%
Rap1a^+/-^ Rap1b^+/-^	76	52	≥100%
Rap1a^+/-^ Rap1b^-/-^	0	26	**0%**
Rap1a^-/-^ Rap1b^+/+^	22	13	≥100%
Rap1a^-/-^ Rap1b^+/-^	3	26	12%
Rap1a^-/-^ Rap1b^-/-^	0	13	**0%**

Number of adult mice from Rap1a+/-, Rap1b+/- intercrosses, at weaning.

Total animal number = 199

*/ Expected number = Total number * Mendelian frequency (1:2:1;2:4:2;1:2:1), based on 100% survival of WT mice.

**Table 3:** Embryonic lethality of double Rap1 KO mice.

Genotype	Total embryo number	Expected */ embryo number	Abnormal (resorbing)	Viable embryos	Viable/Expected
Rap1a^+/+^Rap1b^+/+^	8	8	1	7	92%
Rap1a^+/+^ Rap1b^+/-^	16	15	4	12	79%
Rap1a^+/+^ Rap1b^-/-^	7	8	2	5	66%
Rap1a^+/-^ Rap1b^+/+^	16	15	3	13	85%
Rap1a^+/-^ Rap1b^+/-^	36	31	7	29	95%
Rap1a^+/-^ Rap1b^-/-^	15	15	7	8	52%
Rap1a^-/-^ Rap1b^+/+^	10	8	3	7	92%
Rap1a^-/-^ Rap1b^+/-^	9	15	4	5	33%
Rap1a^-/-^ Rap1b^-/-^	5	8	4	1	13%

Number of E10.5 embryos obtained from Rap1a^+/-^Rap1b^+/-^ intercrosses per genotype.

Total embryo number = 122.

*/Expected number = Total number * Mendelian frequency (1:2:1; 2:4:2; 1:2:1)

### Endothelial-specific double KOs are lethal; either Rap1 isoform is redundant, partial Rap1 deletion leads to partial lethality

Because of the hemorrhaging found in total Rap1-KO embryos (Fig 1) and reduced survival of Rap1-KO mice (Table 2), we hypothesized that vascular defects contribute to lethality in Rap1-KO embryos. To address the role of Rap1 in endothelium during development, we intercrossed Tie2-Cre^+/0^; Rap1a^f/+^, Rap1b^f/+^ mice [33] to generate endothelial-specific Rap1KO (Tie2-Cre^+/0^; Rap1a^f/f^, Rap1b^f/f^, EC-Rap1 KO) mice. Tie2-driven *Cre* recombination leads to efficient Rap1 protein loss in endothelium and hematopoietic cells [33]. We found that deletion of either Rap1b alone (EC-Rap1b KO) or Rap1a alone (EC-Rap1a KO) did not lead to lethality. However, we did not obtain any double Rap1a, Rap1b KO (EC-Rap1 KO) mice (Table 4), similarly to total Rap1-KO mice (Table 2). Interestingly, mice with only one Rap1a allele (EC-Rap1a^+/-^, Rap1b^-/-^) exhibited significantly reduced viability (to 55%) at weaning. In contrast, deletion of all but one Rap1b allele (EC-Rap1a^-/-^, Rap1b^+/-^) minimally impacted mouse survival (Table 4).

**Table 4:** Embryonic lethality of endothelial-specific (EC)-Rap1 KO mice.

Genotype	Total embryo number	Expected */	Abnormal	Viable	Viable/Expected	Comments
Tie2-Cre^+/0^ Rap1a^f/f^ Rap1b^f/f^	2	4.75	2	0	0%	Smaller size, hemorrhage at base of skull
Tie2-Cre^0/0^ Rap1a^f/f^ Rap1b^f/f^	7	4.75	1	6	≥100%	
Tie2-Cre^+/0^ Rap1a^f/f^ Rap1b^f/+^	3	4.75	1	2	42%	Hemorrhage, blood clots, edema
Tie2-Cre^0/0^ Rap1a^f/f^ Rap1b^f/+^	5	4.75	0	5	≥100%	
Tie2-Cre^+/0^ Rap1a^f/+^ Rap1b^f/f^	7	4.75	2	5	≥100%	
Tie2-Cre^0/0^ Rap1a^f/+^ Rap1b^f/f^	7	4.75	1	6	≥100%	
Tie2-Cre^+/0^ Rap1a^f/+^ Rap1b^f/+^	5	4.75	0	5	≥100%	
Tie2-Cre^0/0^ Rap1a^f/+^ Rap1b^f/+^	4	4.75	0	4	84%	

Number of E15.5 embryos obtained from Rap1a^f/f^ Rap1b^f/f^ x Tie2 Cre^+/0^; Rap1a^f/+^ Rap1b ^f/+^ crosses, per genotype.

Total number of embryos = 38.

*/ Expected number = Total number * 1/8 frequency

### Endothelial-specific double KOs suffer from hemorrhage but branchial arch formation appears normal

**Fig 3:**
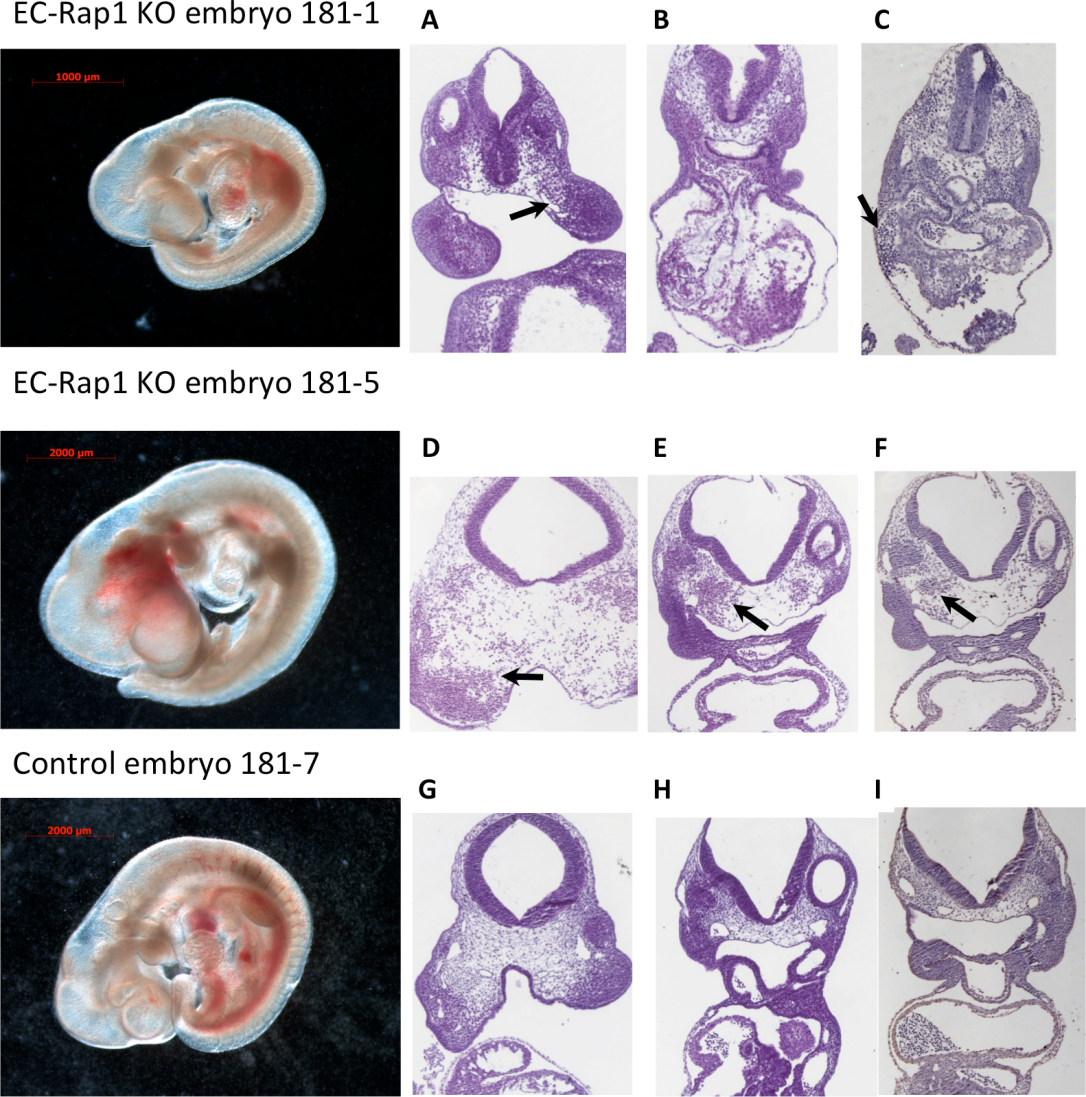
Tissue degeneration and hemorrhages in E10.5 EC-Rap1 KO embryos. Embryos were generated by crossing Tie2-Cre^+/0^ Rap1a ^f/+^ Rap1b^f/+^♂ and Rap1a^f/f^ Rap1b^f/f^♀. Left: whole mount images, left. Right, (A-F): Tissue degeneration (top row) and hemorrhages on the right side of EC-Rap1 KO embryo (middle row), with vascular rapture in or around branchial arches (D-F, arrows) are likely cause of death. Comparable images of control (Tie2-Cre^0^; Rap1a^f/f^, Rap1b^f/f^) embryo (G-I, bottom row).

**Fig 4:**
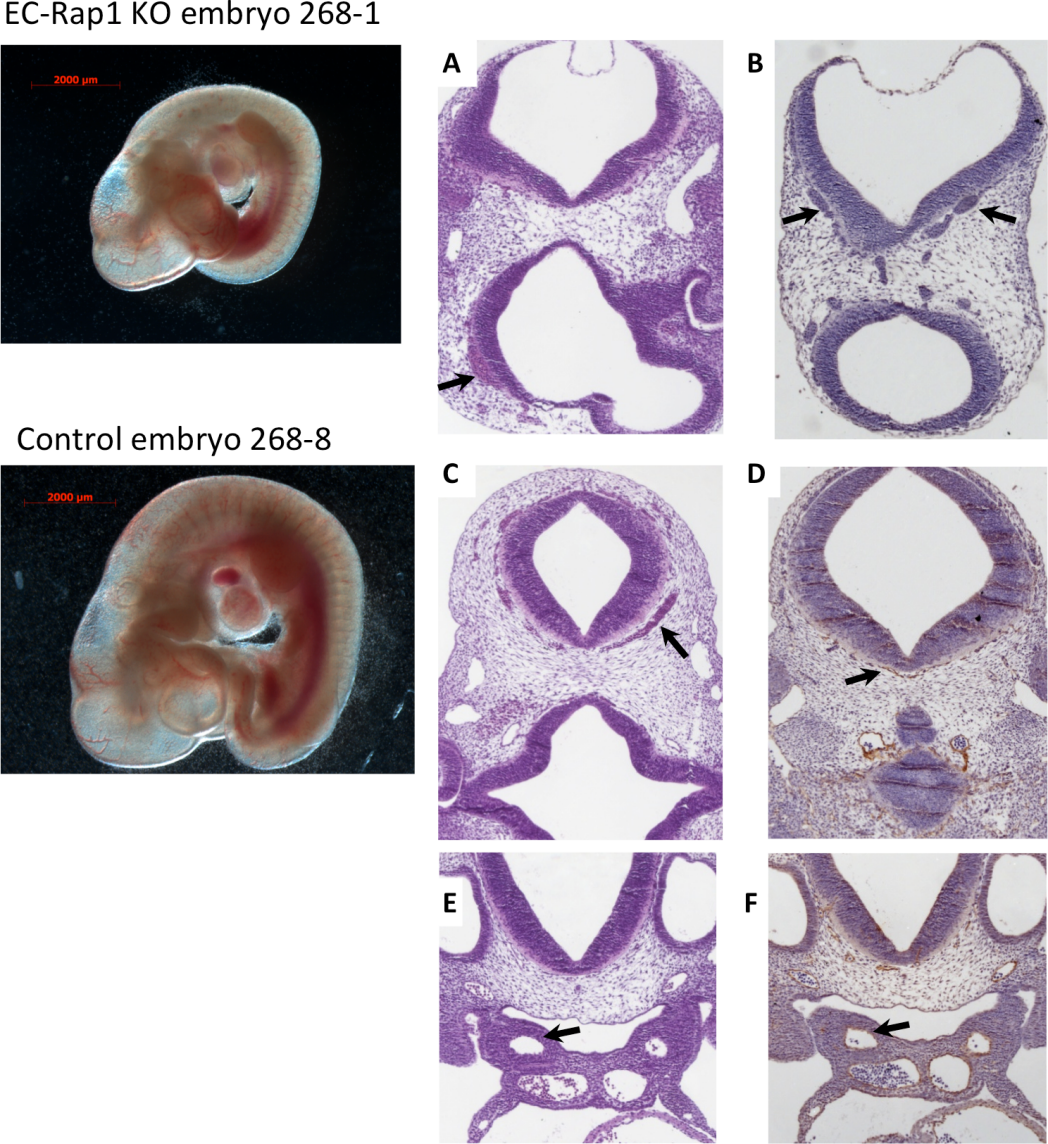
Normal branchial arches in EC-Rap1 KO embryos. Left: whole mount images. Right: (A, B, top row): Dilated microvessels in E10.5 EC-Rap1 KO embryo heads near neural tube (arrows). C-F: Control (Tie2-Cre^0^; Rap1a^f/f^, Rap1b^f/f^, E10.5) embryo sections. Blood-packed perineural vessels (C, D, arrows) are seen in fewer sections than in EC-Rap1KO embryos (B). (E, F, arrows) normal branchial arch arteries. Staining shown is H&E (A, C, E) and CD31 (B, D, F).

**Fig 5:**
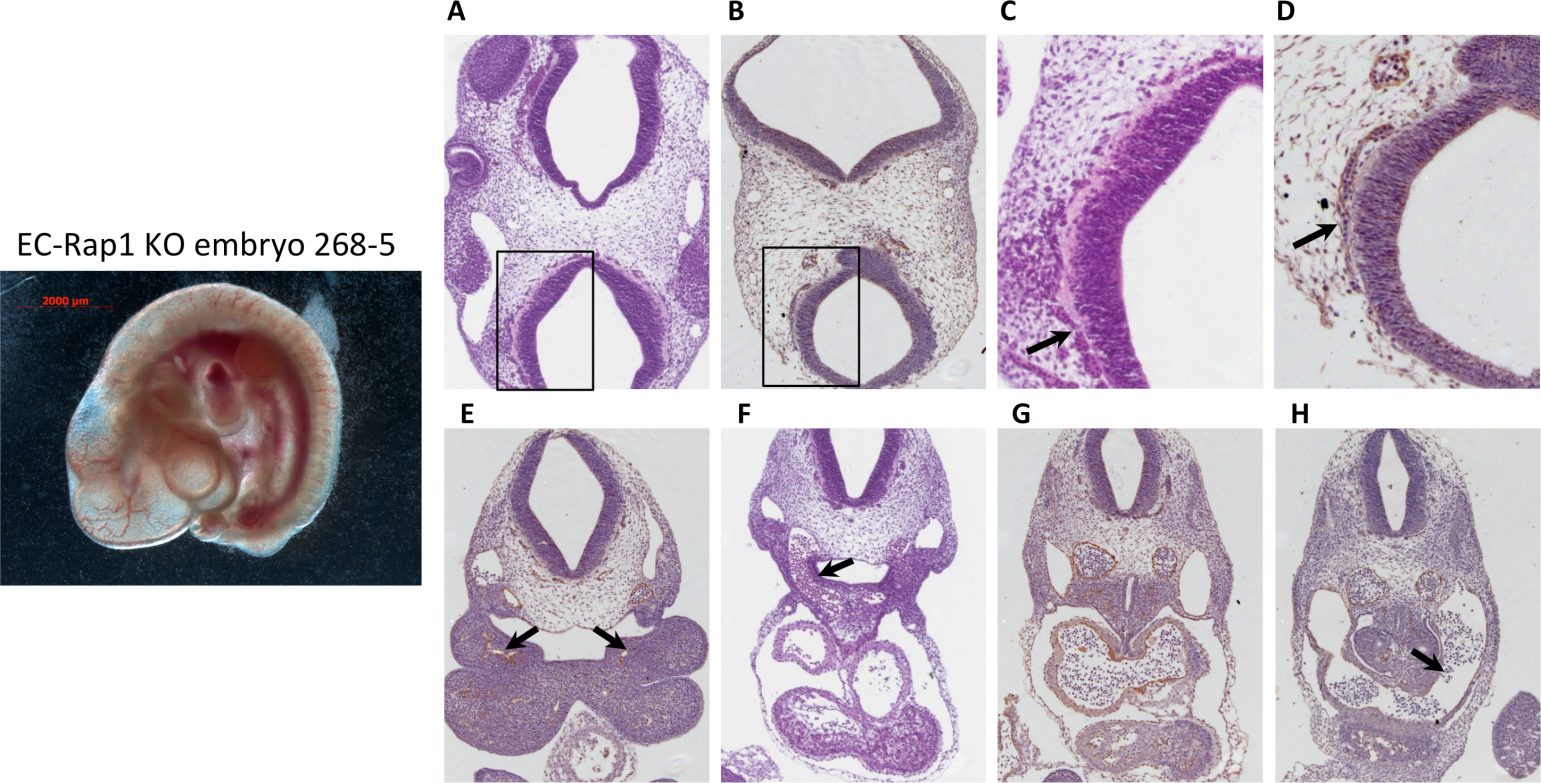
50% of E10.5 EC-Rap1 KO embryos appear grossly normal. Left: whole mount image. Right, A-D: normal perineural vasculature; C and D: enlargement of boxed areas in A and B; (C, arrow) blood-filled vessels; (D, arrow) ECs. E: 1^st^ branchial arch artery, normal (arrow); F: larger 3^rd^ branchial arch artery (arrow); G: aortae and cardinal veins with normal pericardial space at the level of atrium; (H, arrow) sinus venosus. Staining shown is H&E (A, C, F) and CD31 (B, D, E, G and H).

**Fig 6:**
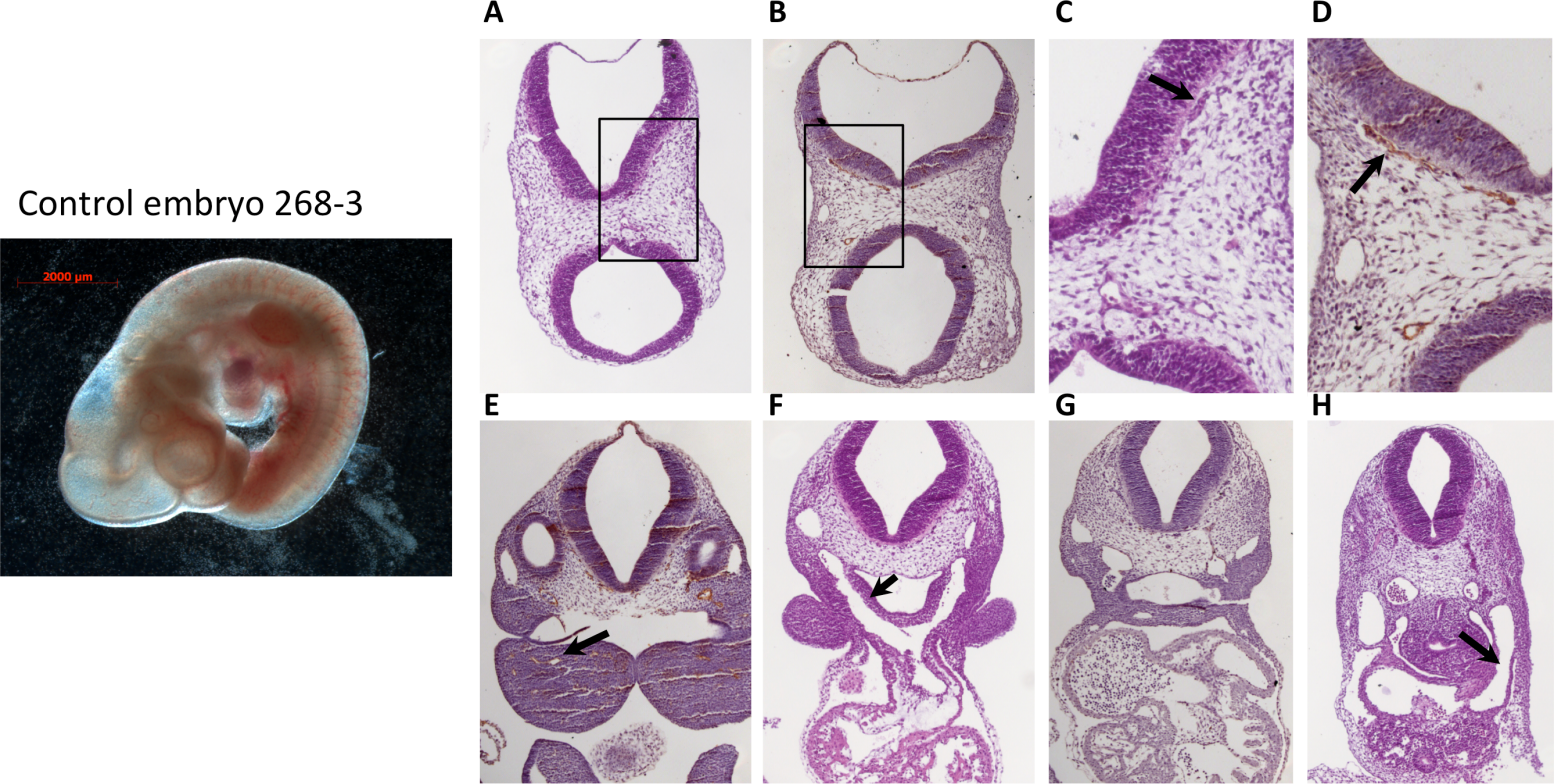
Normal vasculature in control, Tie2-Cre^0/0^; Rap1^f/f^ E10.5 embryos. Left: whole mount image. Right: A-D: Normal perineural vasculature; C and D: enlargement of boxed areas in A and B; (C, arrow) blood-filled vessels; (D, arrow) ECs. (E, arrow) 1^st^ branchial arch artery, normal (arrow); (F, arrow): larger 3^rd^ branchial arch artery; (G): aortae and cardinal veins at the level of arterio-venous canal of the heart, with normal pericardial space; H: venous entrance to the heart (arrow: sinus venosus). Staining shown is H&E (A, C, F and H) and CD31 (B, D, G and H).

To determine the cause of death of EC-Rap1 KO mice, we examined embryos from staged Tie2-Cre^+/0^: Rap1a^f/+^, Rap1b^f/+^ intercrosses. At E10.5 we found that a significant fraction of EC-Rap1 KO embryos were defective compared to WT E10.5 embryos (Figs 3 and 4) with a different embryo size suggestive of different times of death. The defective embryos displayed tissue degeneration (Fig 3A–3F) and cranial hemorrhage on their right side (Fig 3D–3F), with vascular rapture in or around branchial arches as likely cause of death. Furthermore, some EC-Rap1 KO embryos displayed dilated microvessels in the vicinity of the neural tube and vascular engorgement of perineural vessels (Fig 4A and 4B). However, the remaining 50% of EC-Rap1 KO embryos appeared viable without obvious vascular or cardiac lesions (Fig 5; normal EC-Rap1 KO). Interestingly, branchial arch formation appeared normal (Fig 5E and 5F) and similar to WT E10.5 mice (Fig 6E and 6F). This is significantly different from KRIT1 or CCM2 KO mice [35, 36].

**Fig 7:**
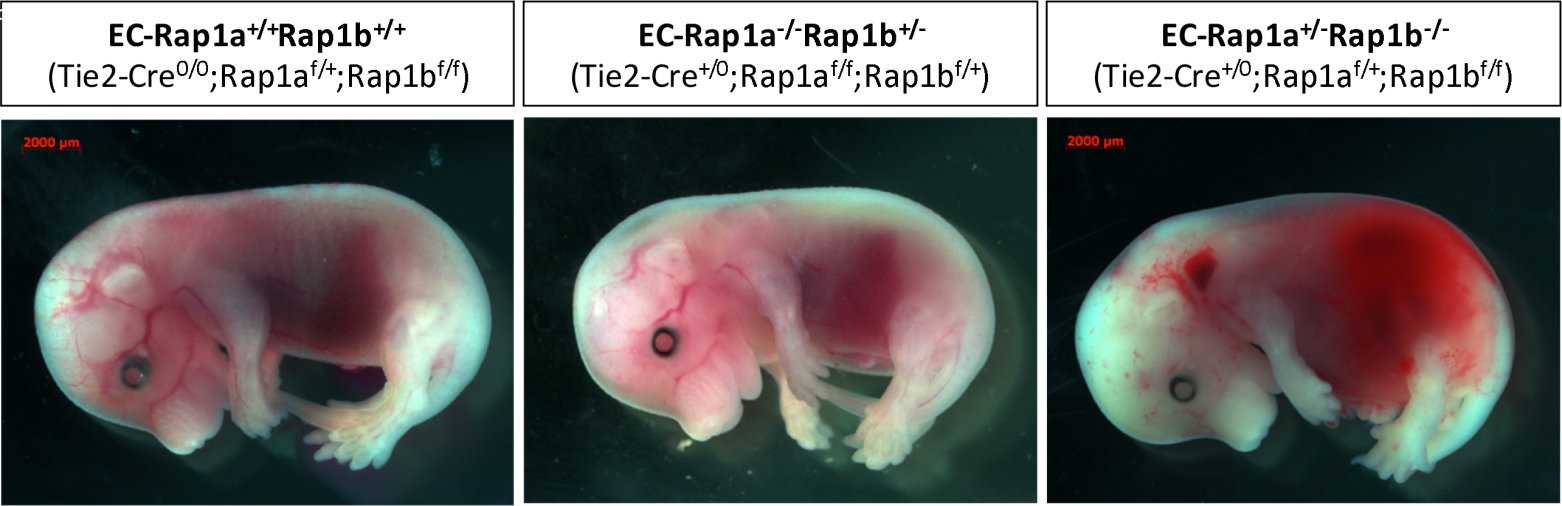
Vascular abnormalities in partial EC-Rap1 KO embryos. Cranial hemorrhage is present in approximately 30% of EC-Rap1a^+/-^Rap1b^-/-^ embryos (right panel) at E15.5 and contributes to increased lethality (44.7%) at weaning. No bleeding was observed in EC-Rap1a^-/-^Rap1b^+/-^ embryos at E15.5 (center). Stereoscopic images are representative of n = 6 analyzed pregnancies resulting from Tie2-Cre^+/0^; Rap1a^f/+^, Rap1b^f/+^ x Tie2-Cre^0/0^; Rap1a^f/f^, Rap1b^f/f^ crosses. Number of animals obtained (N_O_) and expected (N_E_), based on Mendelian distribution, as determined at weaning. Lethality was calculated using the following formula: (1-N_O_/N_E_)*100%.

The analysis at E15.5 did not reveal any viable EC-Rap1 KO embryos (Table 4). Considering the vascular phenotype at E10.5 (Fig 3), death likely resulted from vascular defects. Furthermore, viability of Rap1 mutants containing only one of four Rap1 alleles was reduced (Table 4). We observed that about 30% of E15.5 *EC-Rap1a* ^*+/-*^ *Rap1b* ^*-/-*^ embryos displayed cranial hemorrhage, which contributed to increased lethality (45%) at weaning (Fig 7). No such hemorrhage was observed in *EC-Rap1a* ^*-/-*^ *Rap1b* ^*+/-*^ embryos. Considering that Rap1b is the major Rap1 isoform in ECs in mice (EC-Rap1a^+/-^Rap1b^-/-^ contains less Rap1 protein than EC-Rap1a^-/-^Rap1b^+/-^ mutant) [33], and that neither Rap1 isoform is absolutely required in endothelium for survival (Table 4), this suggests that it may be a quantitative effect and that a certain minimal level of Rap1 is critical for vessel development.

## Discussion

Rap1 is a positive regulator of adhesion and a critical regulator of polarity-dependent morphogenesis in lower organisms [16, 17, 19]. In this paper we demonstrate that Rap1 is essential for early morphogenesis in mice. Deletion of *Rap1a* and *Rap1b* separately has only a small effect on initial development and is not required for embryonic morphogenesis (Table 3). Global deletion of both Rap1 genes leads to major malformation and death before mid-gestation (E10.5). This phenotype is consistent with Rap1 playing a key role in adhesion and polarity *in vivo* also in higher organisms. Furthermore, we demonstrate that Rap1 in endothelium is critically required for vessel formation, as endothelial-specific deletion of both Rap1 isoforms leads to engorgement of perineural vessels, hemorrhage and embryonic lethality between E10.5 and E13.5. Therefore Rap1 is critical for both: tissue and vessel morphogenesis.

Earlier studies in lower organisms demonstrated the importance of the single Rap1 ortholog in tissue morphogenesis through its critical role in the regulation of polarity. Epithelial polarity, required for collective cell migration and tissue morphogenesis, is maintained by cell-cell junctions: tight junctions and cadherin-based adherens junctions, and, physically connected to them, polarized cytoskeleton networks [39]. Rap1 functional interaction with actin cytoskeleton linker Canoe/Afadin is essential to Drosophila morphogenesis [40]. Mechanistically, Rap1 localizes Afadin to cadherin in adherens junctions [19, 41]. Global deletion of Afadin leads to developmental defects during gastrulation and embryonic lethality after E9.5, with loss of structures derived from ectoderm and mesoderm and improper adherens junctions’ organization [42]. The severity of Afadin^-/-^ phenotype exceeds that of Rap1 KO, suggesting the existence of other regulatory mechanisms in the absence of Rap1.

Vessel stability is critical for embryo growth and development. Stabilization of vessels to allow circulation and withstand shear stress forces coincides with dynamic regulation of growth and remodeling. Thus, vascular permeability is tightly regulated and changes in permeability lead to pathologies, including hemorrhage and edema [43]. Our studies show that Rap1 is a critical regulator of vascular stability. Endothelial KO of both Rap1 isoforms leads to localized hemorrhage and tissue degeneration in 50% of double EC-Rap1 KO at mid-gestation. Several Rap1 effectors including KRIT1, RASIP1 and Afadin have been implicated in regulation of vascular permeability by controlling cell-cell junctions. Endothelial deletion of Afadin leads to a defect in postnatal angiogenesis *in vivo* and reduced VEGFR2 signaling [44], similarly to what we reported in EC-Rap1 KO mice [33]. Reduced viability of EC-Afadin KO mice at weaning has been attributed to angiogenesis defect during development [44], however no specific developmental vascular phenotypes have been described. RASIP1, another proposed junctional effector of the EPAC-Rap1 signaling axis, has been implicated in vascular lumen formation [45–47] and more recently, in stabilization of cell-cell junctions required for formation of normal vasculature [48]. RASIP1-KO vessels initially form but are unable to sustain circulation beyond E10.5 in mice, leading to focal hemorrhage and lethality [48]. Similarity of RASIP1 and Afadin KO phenotypes to that of EC-Rap1 KO mice suggests that functional interaction of these molecules is important for murine vascular development. However, approximately half of EC-Rap1 KO embryos are able to form functional vessels and appear grossly normal at mid-gestation (Fig 5). This, again, suggests a regulatory rather than critical role of endothelial Rap1 in early vasculogenesis.

KRIT1 has been implicated as a cell-cell junction target of Rap1 required for vascular stability [6, 49]. Global deletion of KRIT1and CCM2 leads to early and severe vascular pathologies that result in embryo lethality mid-gestation. Vascular defects in KRIT1-null embryos include dilatation of brain vessels and a branchial arch formation defect [35, 50] and endothelial-specific deletion of KRIT1 leads to lethality before E12.5 due to failed vascular development [51]. Global or endothelial-specific CCM2 KO mice die of cardiovascular defects, such as: insufficient vascular lumen formation, defective arteriogenesis and heart malformation [36, 52]. All three CCM genes are essential for embryonic angiogenesis [14, 53, 54]. Interestingly, here we find that EC-Rap1 KO phenotype is distinct from that of KRIT1, as branchial arches form normally in these embryos (Fig 4). Therefore, Rap1 and CCM proteins may not act in the same signaling pathways during development. However, these interactions may still be pertinent in the regulation of cerebral vascular integrity; consequently, their disruption might result in postnatal development of brain hemorrhagic lesions [8, 55].

In addition to directly controlling the function of the above putative effectors, Rap1 may regulate vascular stability via additional mechanisms. We have recently shown that Rap1 is a critical regulator of mechanosensing complexes and shear stress-regulated vessel homeostasis in adult mice [34]. Interestingly, vascular defects observed in endothelial KRIT1-deficiency have also been associated with a defect in endothelial flow response [51]. It is possible that observed EC-Rap1 KO bleeding and embryo lethality might arise from physiologic/hemodynamic abnormality and defective mechanosensing functions, a hypothesis to address in future studies. In addition to the mechanosensing function in endothelium, Rap1b controls vascular tone and blood pressure by limiting smooth muscle contractility [32]. Interestingly, the phenotype of total *Rap1b* ^*-/-*^ mice: hypertension and cardiac hypertrophy, phenocopies that of EC-Rap1a^+/-^Rap1b^-/-^ mice. This suggests that similar hemodynamic defects may contribute to the observed perinatal lethality of pups of the two genotypes.

This study has provided an insight into discrete and redundant functions of two Rap1 isoforms. During initial development, the functions of Rap1a and Rap1b are at least somewhat redundant, as development of the majority of embryos is supported upon single isoform deletion (Table 3) [22, 23]. However, later in development *Rap1b* ^-/-^, but not *Rap1a* ^*-/-*^, embryos develop hemorrhages (Fig 1) and *Rap1a* ^-/-^ embryos develop edema [23](and data not shown). This suggests that the Rap1b isoform may be a more critical regulator of vascular stability during development. While this bleeding phenotype is not observed in EC-Rap1b KO, additional deletion of one Rap1a allele results in a bleeding phenotype similar to that of global Rap1b KO (Fig 7). This suggests that Rap1 in other, non-endothelial cells is required for vessel homeostasis. Such a conclusion is supported by our findings of the importance of Rap1b in smooth muscle cells in maintenance of vascular tone [32]. Thus, additional studies of Rap1 in other vascular cells are required for full understanding of the role of this important molecule in vessel homeostasis.
